# Cumulative exposure to the remnant cholesterol inflammation index and incident diabetes mellitus in middle-aged and elderly Chinese adults: A nationwide cohort study from CHARLS

**DOI:** 10.1097/MD.0000000000051063

**Published:** 2026-10-09

**Authors:** Lianguang Xie, Xuan Zhou, Chun Yao, Diankui Shui

**Affiliations:** a Liuzhou Traditional Chinese Medical Hospital, Liuzhou, Guangxi, China; b Department of Public Health, School of Medicine, Guangxi University of Science and Technology, Liuzhou, Guangxi, China; c Guangxi University of Chinese Medicine, Nanning, Guangxi, China.

**Keywords:** CHARLS, cumulative exposure, diabetes mellitus, inflammation, remnant cholesterol inflammation index

## Abstract

The remnant cholesterol inflammation index (RCII) is a novel biomarker integrating remnant cholesterol and high-sensitivity C-reactive protein. Whether long-term exposure to RCII is associated with diabetes mellitus (DM) remains unclear. We examined the association between cumulative RCII (cumRCII) exposure and incident DM risk in the China Health and Retirement Longitudinal Study. We included 5817 participants with DM at baseline from the China Health and Retirement Longitudinal Study 2011 to 2015. Logistic regression models and restricted cubic splines were used to examine the relationship between cumRCII and DM risk, adjusting for potential confounding factors. Subgroup analysis was used to examine potential effect modification, and sensitivity analysis was used to test the robustness of the primary findings. A total of 786 new-onset DM cases occurred. Participants in the highest quartile of cumRCII levels had a higher DM risk than those in the lowest quartile (odds ratio = 2.47; 95% confidence interval = 1.95–3.12; *P*_trend_ < .001). Each 1-SD increment in cumRCII was associated with a 25% increased risk (odds ratio = 1.25; 95% confidence interval = 1.18–1.33). Restricted cubic spline analysis indicated a positive and nonlinear association between cumRCII and DM (*P*_for overall_ < .001; *P*_for nonlinearity_ < .001). Subgroup analysis revealed consistent associations between cumRCII and DM risk (all *P*_for interaction_ > .05). *E*-value analysis suggested robustness to unmeasured confounding. Elevated long-term cumRCII is significantly associated with higher DM risk in middle-aged and elderly Chinese adults, suggesting that cumRCII may serve as a potential biomarker for identifying individuals at higher metabolic risk.

## 1. Introduction

Diabetes mellitus (DM) has become a major global public health challenge, and there will be 853 million DM patients worldwide among adults by 2050.^[1]^ DM incidence continues to rise among the elderly in China.^[2]^ China currently has one of the world’s largest diabetes populations, accounting for about a quarter of global cases.^[3]^ DM is one of the main causes of death and disability, which affects people’s physical and mental health.^[4]^ Exploring effective prevention and control strategies for DM holds significant practical importance for addressing China’s aging population trend.

Accumulating evidence suggests that lipid metabolism disorders and chronic inflammation play pivotal roles in the pathogenesis of DM.^[5–7]^ Inflammation can directly interfere with the insulin signal transduction pathway and have toxic effects on islet β cells, ultimately leading to an imbalance in glucose metabolism homeostasis.^[5]^ High-sensitivity C-reactive protein (hsCRP) is recognized as a key biomarker of inflammatory response. Increased hsCRP levels are not only significantly associated with an increased risk of DM^[8]^ but also strongly linked to chronic complications of DM, such as diabetic nephropathy^[9,10]^ and diabetic neuropathy.^[11,12]^ Beyond traditional risk factors, remnant cholesterol (RC) is independent of low-density lipoprotein and can be used as a supplement for the pathological process of DM.^[13]^ RC consists mainly of triglyceride (TG)-rich lipoproteins, which are lipid components reflecting TG-rich lipoproteins.^[14]^ Research indicates that even after achieving the guideline-recommended targets for low-density lipoprotein cholesterol (LDL-C), many patients still face residual risk challenges.^[15]^ Recent studies have shown that RC may be associated with the risk of cardiovascular disease,^[16]^ stroke,^[17]^ depression,^[18]^ prediabetes ^[19]^ and DM.^[13]^It has been found that RC may induce a systemic low-grade inflammatory environment characterized by elevated CRP levels through leukocyte activation.^[20]^ High RC levels have been associated with higher hsCRP levels in studies, suggesting that elevated RC is associated with an increased systemic inflammatory state.^[21,22]^ However, the specific impact of RC and CRP combinations and cumulative effects on DM risk is not fully understood, and further research is needed.

Recent studies have shown that integrating lipid metabolism and inflammatory markers is associated with cardiovascular disease risk in populations and can enhance the predictive power of cardiovascular risk models.^[23]^ Remnant cholesterol inflammation index (RCII) is a novel composite biomarker that multiplies RC and hsCRP levels to capture pathophysiological risks driven by lipid metabolism disorders and chronic inflammation simultaneously.^[24]^ RCII has been shown to have potential prognostic significance in cardiovascular disease,^[24]^ is associated with all-cause mortality and specific mortality in the elderly,^[25]^ and is also associated with stroke risk in the population.^[26,27]^ However, there is still a lack of association studies between RCII and the risk of new-onset DM. Evidence showed that the RCII may serve as a biomarker for predicting all-cause mortality and cardiovascular disease risks in individuals with prediabetes or DM.^[24]^ Moreover, prior investigations have focused on single-point RCII assessments, which is insufficient evidence from longitudinal cumulative effects of RCII for metabolic–inflammatory burden. The association of cumulative RCII (cumRCII) with DM risk was uncertain.

Therefore, in this study, we estimated the association between baseline and long-term cumulative exposure of RCII and the risks of DM incident, using the China Health and Retirement Longitudinal Study (CHARLS) data from 2011 to 2015.

## 2. Materials and methods

### 2.1. Study design and population

All data were sourced from the CHARLS cohort, a widely recognized longitudinal study that focuses on individuals middle-aged and older (≥45 years). The initial nationwide survey of CHARLS was conducted in 2011 (wave 1), successfully interviewing 17,708 individuals. The study commenced with a baseline survey, after which follow-up assessments were conducted at 2-year intervals. Specifically, waves 2, 3, and 4 were completed in the years 2013, 2015, and 2018, respectively. Blood samples were also collected from participants during wave 1 and wave 3, with corresponding sample sizes of 11,847 and 13,420 individuals. Comprehensive details regarding the sampling methodology, anthropometric measurements, and blood-based biomarker data are directed to previously published literature.^[28,29]^ Considering available blood examination data, the datasets for wave 1 (baseline, 2011) and wave 3 (follow-up, 2015) were extracted.

We first included 17,708 participants at wave 1, and we excluded those without blood sample (n = 5861), without demographic data (n = 97), without follow-up visits at wave 3 (n = 1399), or without complete data on total cholesterol (TC), high-density lipoprotein cholesterol (HDL-C), LDL-C, CRP, TG, or fasting blood glucose (FBG) at wave 1 or wave 3 (n = 2898). Participants younger than 45 years old (n = 225), participants with DM at wave 1 (n = 1339), and participants with extreme values of cumRCII (less or more than 3 standard deviations from the mean; n = 72) were excluded. The rationale for applying the 3 standard deviations threshold was to minimize the potential impact of data entry errors or extreme measurement outliers on the stability of the regression models. Ultimately, a total of 5817 participants remained in the final study (Fig. 1). The ethical approval process of CHARLS was conducted by the Peking University Biomedical Ethics Review Committee (IRB00001052-11015). All participants provided formal written consent.

**Figure 1.**
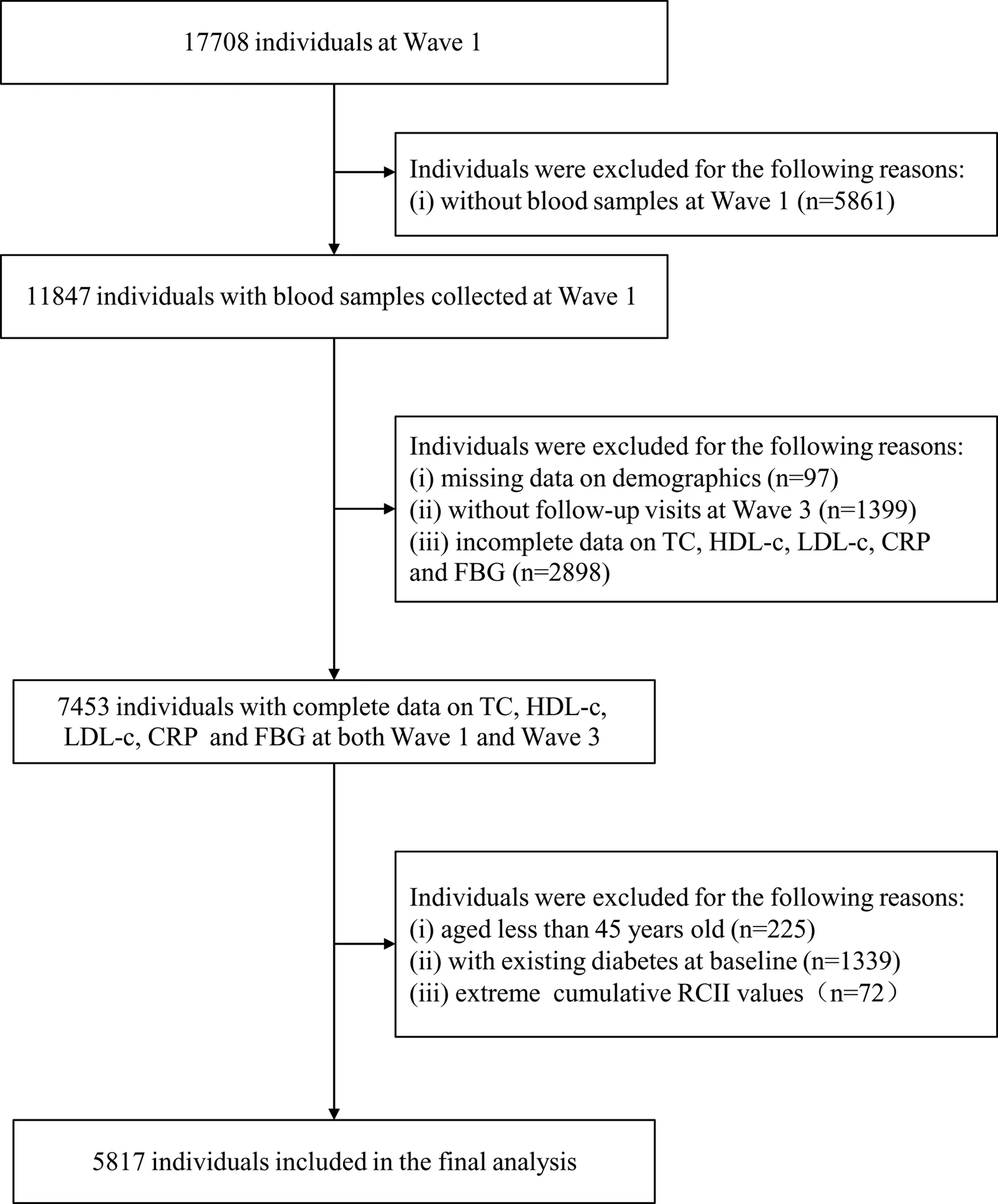
The flowchart of this study. CRP = C-reactive protein, FBG = fasting blood glucose, HDL-C = high-density lipoprotein cholesterol, LDL-C = low-density lipoprotein cholesterol, RCII = remnant cholesterol inflammation index, TC = total cholesterol.

### 2.2. Assessment of RCII and cumRCII

RC was computed as follows: RC (mg/dL) = TC−(HDL-C + LDL-C). RCII at each wave was RC multiplied by hsCRP (mg/L): RCII = RC (mg/dL) × hsCRP (mg/L)/10.^[27]^ CumRCII was calculated with the trapezoidal rule: [(RCII_2011_ + RCII_2015_)/2] × 4 years.

### 2.3. Assessment of DM

The new-onset DM was defined based on self-report, laboratory tests, and medical history: respondents answered “yes” to the interviewer’s question about DM status, “Have you been diagnosed with diabetes by a doctor?” or FBG ≥ 126 mg/dL and/or glycosylated hemoglobin (HbA1c) ≥ 6.5%, or use of antidiabetic medications.

### 2.4. Covariates

Covariates were collected on sociodemographic characteristics (age, gender, education level, residence status, marital status, and body mass index [BMI]), systolic blood pressure, diastolic blood pressure, and lifestyle (smoking status and drinking status). In addition, participants’ medical histories were recorded, including dyslipidemia, hypertension, heart disease, kidney disease, liver disease, and DM. Laboratory-based biomarkers were also measured, comprising HbA1c, FBG, hsCRP, TGs, TC, HDL-C, and LDL-C.

### 2.5. Statistical analysis

Continuous data conforming to a normal distribution are summarized as mean ± standard deviation, whereas those deviating from normality are expressed as median and interquartile range. For categorical variables, frequency counts and corresponding percentages are reported. Inter-group comparisons of baseline characteristics were conducted using appropriate statistical tests: continuous measures were analyzed with either one-way analysis of variance (for normally distributed data) or the Kruskal–Wallis test (for non-normal data), while associations involving categorical variables were assessed with the Pearson chi-square test. A multiple imputation procedure was employed. The missingness rates for the main covariates ranged from 0.10% (drinking status) to 11.78% (BMI). We assumed that the data were missing at random, and this assumption is plausible given the detailed questionnaire and medical history data available in CHARLS. We generated 5 imputed datasets and pooled estimates using Rubin rules to account for uncertainty. The imputation model used linear regression (continuous variables) or logistic regression (categorical variables) with 50 iterations and included all variables in the analysis model. The imputation of each variable utilizes all other variables except itself as predictor variables. Diagnostic assessments of imputation quality used convergence diagnostics and density plots.

Given CHARLS only contains 2 waves with uniformly fixed 4-year follow-up and interval-censored DM onset information, univariable and multivariable logistic regression was adopted to evaluate the relationship between cumRCII and DM without introducing artificial precision associated with arbitrary survival time assignments. The strength of these associations is reported as odds ratio (OR) alongside their 95% confidence interval (CI). A sequence of 4 statistical models was developed with progressive adjustments for confounding factors: the crude model (model 1) included no covariates; model 2 controlled for age and gender; model 3 incorporated additional sociodemographic and lifestyle variables, namely education level, marital status, smoking status, drinking status, residence, and BMI; model 4 further extended the adjustment to include clinical comorbidities such as hypertension, dyslipidemia, and histories of liver, kidney, and heart disease.

Restricted cubic spline (RCS) analysis was implemented within a fully adjusted logistic regression to explore the nonlinear association between cumRCII and DM risk. This model incorporated 4 knots, positioned at the 5th, 35th, 65th, and 95th percentiles of the cumRCII distribution. In addition, potential effect modification was assessed through stratified subgroup analyses. These analyses utilized logistic regression models to examine the relationship within predefined strata based on demographic factors (age, gender, education, marital status, residence), behavioral factors (smoking, alcohol consumption), anthropometric measures (BMI), and clinical comorbidities (hypertension, dyslipidemia, liver, kidney, and heart disease). To test the robustness of the primary findings, sensitivity analyses were carried out: repeating the analysis on a complete-case dataset, excluding individuals with any missing covariate data; rerunning the analysis after removing participants who had already been diagnosed with DM at wave 2 (2013); reanalyzing the data after excluding prediabetic individuals at wave 1 (2011) based on FBG (100–125 mg/dL) or HbA1c (5.7%–6.4%); reanalyzing including participants with extreme values of cumRCII (less or more than 3 standard deviations from the mean); and *E*-value was calculated to quantify the effect of potential unmeasured confounders on the results.^[30,31]^ All tests were carried out using R 4.3.0 statistical software (R Foundation for Statistical Computing). Differences were considered statistically significant at a two-tailed *P* value < .05.

## 3. Results

### 3.1. Baseline characteristics of participants

A total of 5817 individuals were analyzed. A total of 786 participants developed DM (incidence rate 13.51%). The mean age was 58.44 ± 8.73 years, with 46.26% male (2691) and 53.74% female (3126) participants. The mean RCII value was 4.69 ± 9.35, and the cumRCII was 18.17 ± 24.49. Participants were categorized into 4 groups (Q1–Q4) based on cumRCII. Compared to those in the lowest quartile (Q1), participants in the highest quartile (Q4) were older and had higher BMI, systolic blood pressure, diastolic blood pressure, FBG, and TG levels, while exhibiting significantly lower HDL-C levels (all *P*_for trend_ < .001). Furthermore, the prevalence of hypertension, DM, and dyslipidemia increased significantly across ascending cumRCII quartiles (all *P* < .001). Regarding lifestyle factors, a significant difference in smoking status was observed across the cumRCII groups (*P* < .001). No significant differences were found in the distribution of gender, drinking status, or education level among the groups (Table 1). The baseline characteristics of included and excluded participants were presented in Table S1, Supplemental Digital Content 1.

**Table 1:** Characteristics of participants stratified by cumulative RCII quartiles.

Variables	Total (n = 5817)	Q1 (n = 1455)	Q2 (n = 1454)	Q3 (n = 1454)	Q4 (n = 1454)	*P* value
Age, yr	58.44 ± 8.73	57.84 ± 8.63	58.48 ± 8.61	58.44 ± 8.89	59.00 ± 8.78	**.005**
BMI, kg/m^2^	24.18 ± 36.24	22.07 ± 3.14	23.18 ± 3.89	26.85 ± 71.76	24.56 ± 4.11	**.006**
SBP, mm Hg	128.08 ± 21.00	124.18 ± 20.13	126.62 ± 20.55	129.34 ± 20.24	132.13 ± 22.18	**<.001**
DBP, mm Hg	74.95 ± 12.23	72.67 ± 11.89	73.90 ± 12.12	75.93 ± 12.11	77.27 ± 12.29	**<.001**
HbA1c, %	5.10 ± 0.39	5.06 ± 0.39	5.09 ± 0.39	5.10 ± 0.39	5.13 ± 0.40	**<.001**
FBG, mg/dL	100.01 ± 11.58	98.48 ± 11.15	99.69 ± 11.50	100.21 ± 11.57	101.67 ± 11.86	**<.001**
TG, mg/dL	120.95 ± 73.64	84.87 ± 38.05	105.20 ± 47.84	128.56 ± 69.50	165.19 ± 97.83	**<.001**
TC, mg/dL	192.04 ± 36.84	184.42 ± 33.66	190.37 ± 36.36	193.09 ± 36.35	200.28 ± 39.05	**<.001**
HDL-C, mg/dL	52.10 ± 14.94	58.31 ± 14.82	53.86 ± 14.31	50.28 ± 14.01	45.95 ± 13.80	**<.001**
LDL-C, mg/dL	116.88 ± 33.44	113.16 ± 29.92	117.48 ± 32.67	117.79 ± 33.78	119.09 ± 36.76	.441
CRP, mg/L	2.11 ± 4.62	0.65 ± 1.06	1.03 ± 0.95	1.75 ± 1.87	5.02 ± 8.25	**<.001**
RC, mg/dL	23.06 ± 18.96	12.95 ± 9.70	19.03 ± 12.50	25.02 ± 16.72	35.24 ± 25.27	**<.001**
RCII	4.69 ± 9.35	0.66 ± 0.58	1.60 ± 1.10	3.58 ± 2.46	12.94 ± 15.72	**<.001**
CumRCII	18.17 ± 24.49	2.72 ± 1.12	6.81 ± 1.45	14.10 ± 3.17	49.06 ± 32.36	**<.001**
Smoking status, n (%)						.321
Never smoked	1731 (30.16)	443 (30.96)	441 (30.77)	417 (29.02)	876 (60.92)	
Former smoker	458 (7.98)	97 (6.78)	115 (8.03)	114 (7.93)	132 (9.18)	
Current smoker	3550 (61.86)	891 (62.26)	877 (61.20)	906 (63.05)	430 (29.90)	
Drinking status, n (%)						**.025**
Never drinker	1807 (31.10)	490 (33.77)	471 (32.42)	429 (29.53)	914 (62.86)	
Former drinker	449 (7.73)	95 (6.55)	118 (8.12)	113 (7.78)	123 (8.46)	
Current drinker	3555 (61.18)	866 (59.68)	864 (59.46)	911 (62.70)	417 (28.68)	
Education level, n (%)						.695
College or above	155 (2.66)	34 (2.34)	40 (2.75)	38 (2.61)	43 (2.96)	
Elementary school or below	4101 (70.50)	1014 (69.69)	1033 (71.05)	1014 (69.74)	1040 (71.53)	
Middle school	1561 (26.84)	407 (27.97)	381 (26.20)	402 (27.65)	371 (25.52)	
Gender, n (%)						.132
Male	2691 (46.26)	702 (48.25)	687 (47.25)	656 (45.12)	646 (44.43)	
Female	3126 (53.74)	753 (51.75)	767 (52.75)	798 (54.88)	808 (55.57)	
Marital status, n (%)						.180
Others	872 (14.99)	218 (14.98)	214 (14.72)	199 (13.69)	241 (16.57)	
Married	4945 (85.01)	1237 (85.02)	1240 (85.28)	1255 (86.31)	1213 (83.43)	
Residence status, n (%)						**.014**
Urban	895 (15.39)	207 (14.23)	201 (13.82)	229 (15.75)	258 (17.74)	
Rural	4922 (84.61)	1248 (85.77)	1253 (86.18)	1225 (84.25)	1196 (82.26)	
Dyslipidemia, n (%)						**<.001**
No	5231 (91.31)	1364 (95.38)	1322 (92.13)	1308 (91.21)	1237 (86.50)	
Yes	498 (8.69)	66 (4.62)	113 (7.87)	126 (8.79)	193 (13.50)	
Hypertension, n (%)						**<.001**
No	4377 (75.56)	1207 (83.41)	1156 (79.83)	1046 (72.09)	968 (66.90)	
Yes	1416 (24.44)	240 (16.59)	292 (20.17)	405 (27.91)	479 (33.10)	
Kidney disease, n (%)						.404
No	5448 (94.17)	1365 (94.40)	1370 (94.68)	1364 (94.33)	1349 (93.29)	
Yes	337 (5.83)	81 (5.60)	77 (5.32)	82 (5.67)	97 (6.71)	
Liver disease, n (%)						.141
No	5597 (96.70)	1397 (96.75)	1396 (96.34)	1396 (96.14)	1408 (97.57)	
Yes	191 (3.30)	47 (3.25)	53 (3.66)	56 (3.86)	35 (2.43)	
Heart disease, n (%)						**<.001**
No	5115 (88.28)	1322 (91.30)	1299 (89.46)	1259 (86.83)	1235 (85.53)	
Yes	679 (11.72)	126 (8.70)	153 (10.54)	191 (13.17)	209 (14.47)	
Diabetes, n (%)						**<.001**
No	5031 (86.49)	1340 (92.10)	1280 (88.03)	1241 (85.35)	1170 (80.47)	
Yes	786 (13.51)	115 (7.90)	174 (11.97)	213 (14.65)	284 (19.53)	

Bold values indicate statistically significant differences.

BMI = body mass index, CRP = C-reactive protein, cumRCII = cumulative remnant cholesterol inflammation index, DBP = diastolic blood pressure, FBG = fasting blood glucose, HbA1c = glycosylated hemoglobin A1c, HDL-C = high‐density lipoprotein cholesterol, LDL-C = low-density lipoprotein cholesterol, RC = remnant cholesterol, RCII = remnant cholesterol inflammation index, SBP = systolic blood pressure, TC = total cholesterol, TG = triglyceride.

### 3.2. Association between the RCII, cumRCII, and DM incidence

In the crude model, each SD increase in RCII and cumRCII was associated with a 15% and 30% higher risk of DM, respectively (OR = 1.15, 95% CI = 1.09–1.23; OR = 1.30, 95% CI = 1.22–1.38). Participants in the highest quartile (Q4) had 99% and 183% higher risks of DM compared to those in the lowest quartile (Q1) for RCII and cumRCII, respectively. Specifically, the fully adjusted ORs per SD increase were 1.13 (95% CI = 1.06–1.20) for RCII and 1.25 (95% CI = 1.18–1.33) for cumRCII. Similarly, in the fully adjusted model, the risks in Q4 remained significantly elevated compared to Q1 (RCII Q4: OR = 1.74, 95% CI = 1.39–2.18; cumRCII Q4: OR = 2.47, 95% CI = 1.95–3.12), with significant trend tests (*P*_for trend_ < .001; Table 2).

**Table 2:** Association between the RCII, cumulative RCII, and DM incidence.

Variables	Model 1		Model 2		Model 3		Model 4	
	OR (95% CI)	*P* value	OR (95% CI)	*P* value	OR (95% CI)	*P* value	OR (95% CI)	*P* value
RCII								
Per SD increase	1.15 (1.09–1.23)	**<.001**	1.15 (1.08–1.22)	**<.001**	1.15 (1.08–1.22)	**<.001**	1.13 (1.06–1.20)	**<.001**
Quartiles								
Q1	1.00 (reference)		1.00 (reference)		1.00 (reference)		1.00 (reference)	
Q2	1.27 (1.01–1.61)	**.048**	1.25 (0.99–1.58)	.064	1.25 (0.99–1.58)	.064	1.21 (0.96–1.54)	.109
Q3	1.67 (1.33–2.09)	**<.001**	1.62 (1.29–2.04)	**<.001**	1.62 (1.29–2.04)	**<.001**	1.49 (1.19–1.88)	**<.001**
Q4	1.99 (1.59–2.48)	**<.001**	1.92 (1.54–2.40)	**<.001**	1.92 (1.54–2.40)	**<.001**	1.74 (1.39–2.18)	**<.001**
*P* for trend		**<.001**		**<.001**		**<.001**		**<.001**
CumRCII								
Per SD increase	1.30 (1.22–1.38)	**<.001**	1.29 (1.21–1.37)	**<.001**	1.28 (1.21–1.36)	**<.001**	1.25 (1.18–1.33)	**<.001**
Quartiles								
Q1	1.00 (reference)		1.00 (reference)		1.00 (reference)		1.00 (reference)	
Q2	1.58 (1.24–2.03)	**<.001**	1.56 (1.22–2.01)	**<.001**	1.56 (1.22–2.00)	**<.001**	1.52 (1.18–1.95)	**.001**
Q3	2.00 (1.57–2.54)	**<.001**	1.97 (1.55–2.51)	**<.001**	1.96 (1.54–2.50)	**<.001**	1.84 (1.45–2.35)	**<.001**
Q4	2.83 (2.25–3.56)	**<.001**	2.76 (2.19–3.48)	**<.001**	2.74 (2.17–3.45)	**<.001**	2.47 (1.95–3.12)	**<.001**
*P*_for trend_		**<.001**		**<.001**		**<.001**		**<.001**

Model 1: crude.

Model 2: adjusted for age, gender.

Model 3: adjusted for age, gender, education level, marital status, smoking status, drinking status, residence, BMI.

Model 4: adjusted for age, gender, education level, marital status, smoking status, drinking status, residence, BMI, hypertension, dyslipidemia, liver disease, kidney disease, heart disease.

Bold values indicate statistically significant differences.

BMI = body mass index, CI = confidence interval, cumRCII = cumulative remnant cholesterol inflammation index, DM = diabetes mellitus, OR = odds ratio, RCII = remnant cholesterol inflammation index, SD = standard deviation.

### 3.3. RCS analysis

The fully adjusted RCS regression model demonstrated a significant positive and nonlinear association between RCII, cumRCII, and the risk of DM (all *P*_for overall_ < .001; all *P*_for nonlinearity_ < .001; Figs. 2 and 3). The cutoff for RCII is 7.106, and for cumRCII, it is 21.767.

**Figure 2.**
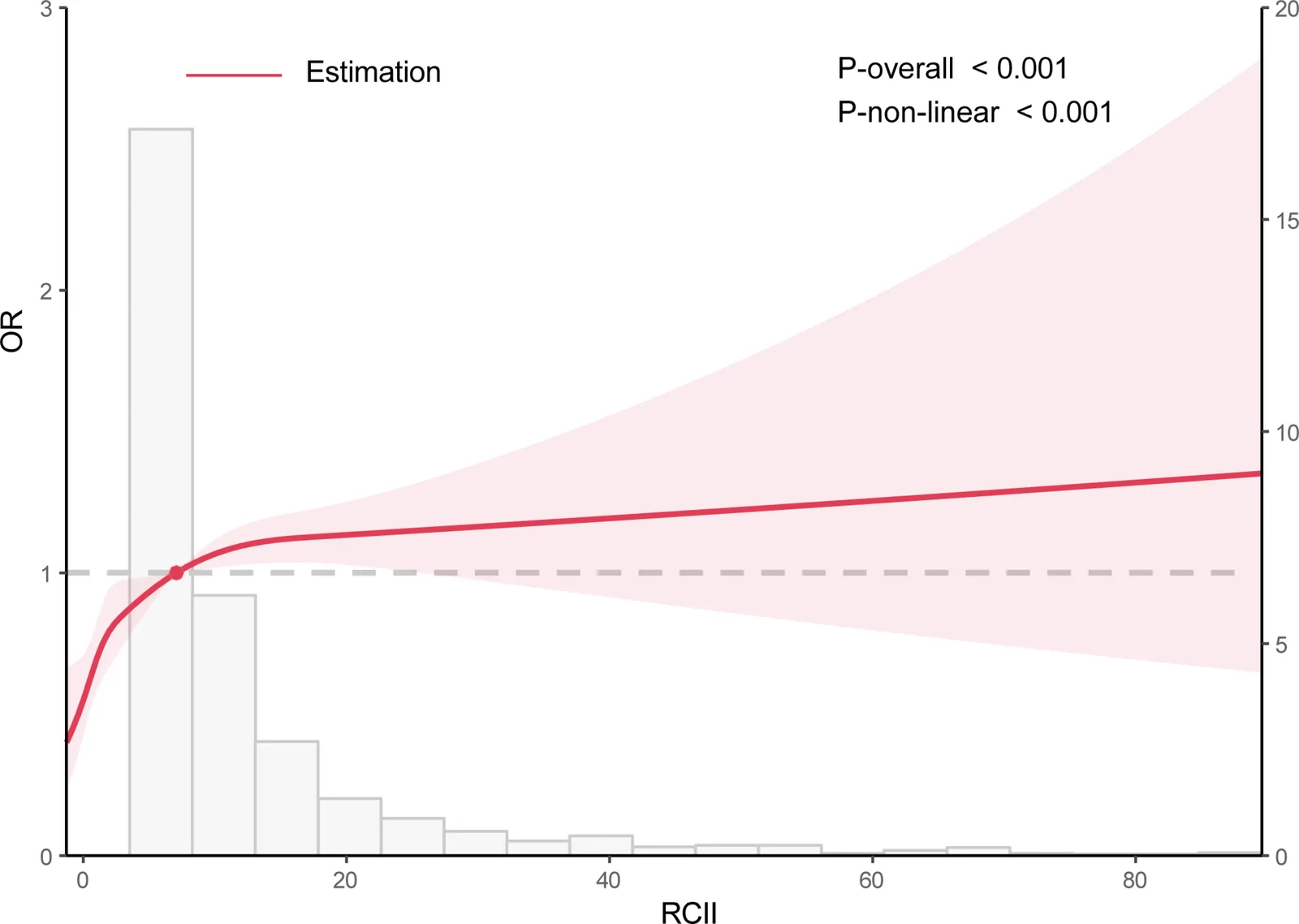
RCS curve between RCII and DM incidence. DM = diabetes mellitus, OR = odds ratio, RCII = remnant cholesterol inflammation index, RCS = restricted cubic spline.

**Figure 3.**
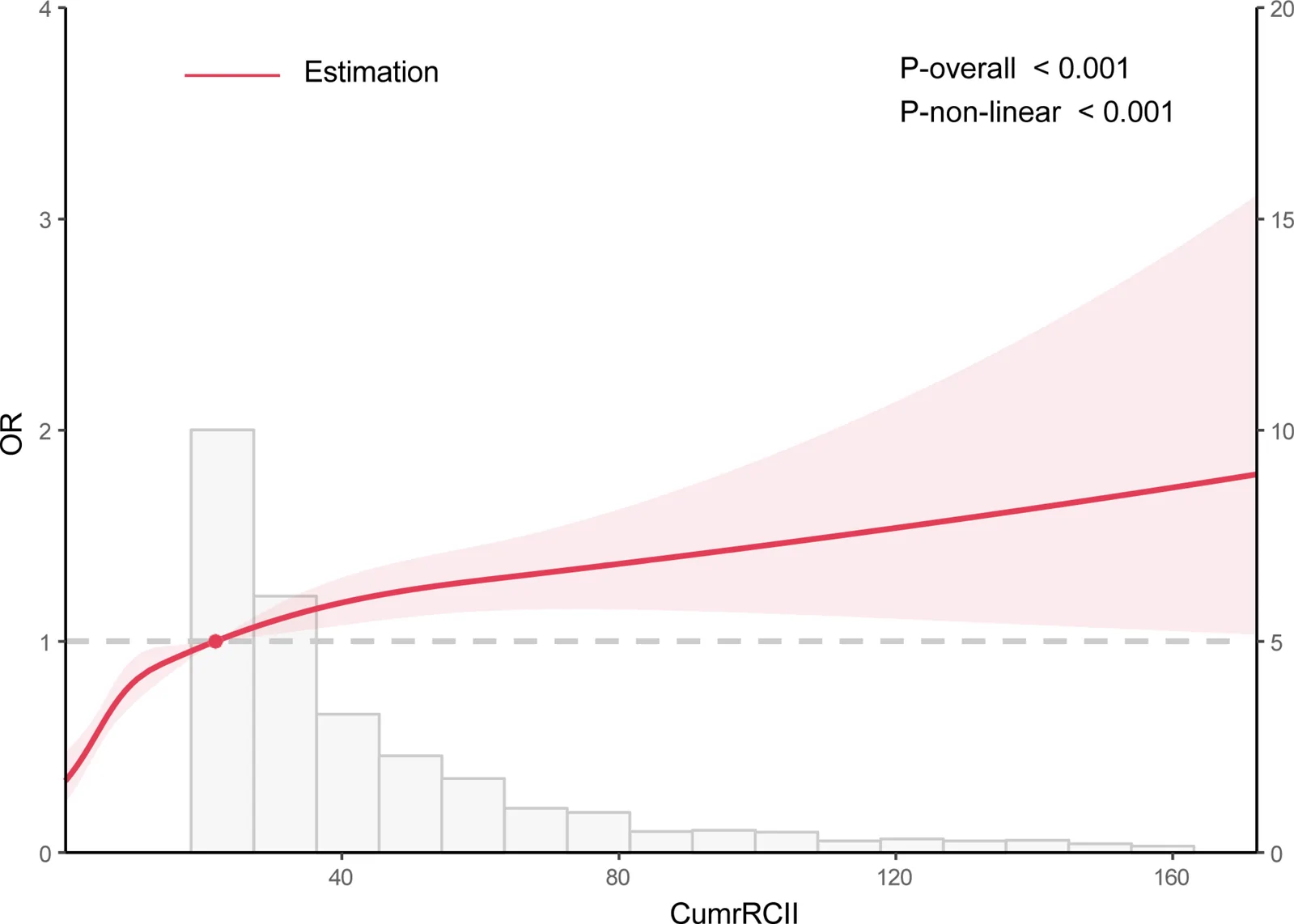
RCS curve between cumRCII and DM incidence. cumRCII = cumulative remnant cholesterol inflammation index, DM = diabetes mellitus, OR = odds ratio, RCS = restricted cubic spline.

### 3.4. Subgroup analysis

As shown in Figure 4, no significant interactions were observed for age, gender, current smoker, current drinker, obesity, education, marital status, residence status, and prevalence of dyslipidemia, hypertension, kidney disease, liver disease, or heart disease strata (all *P*_for interaction_ > .05).

**Figure 4.**
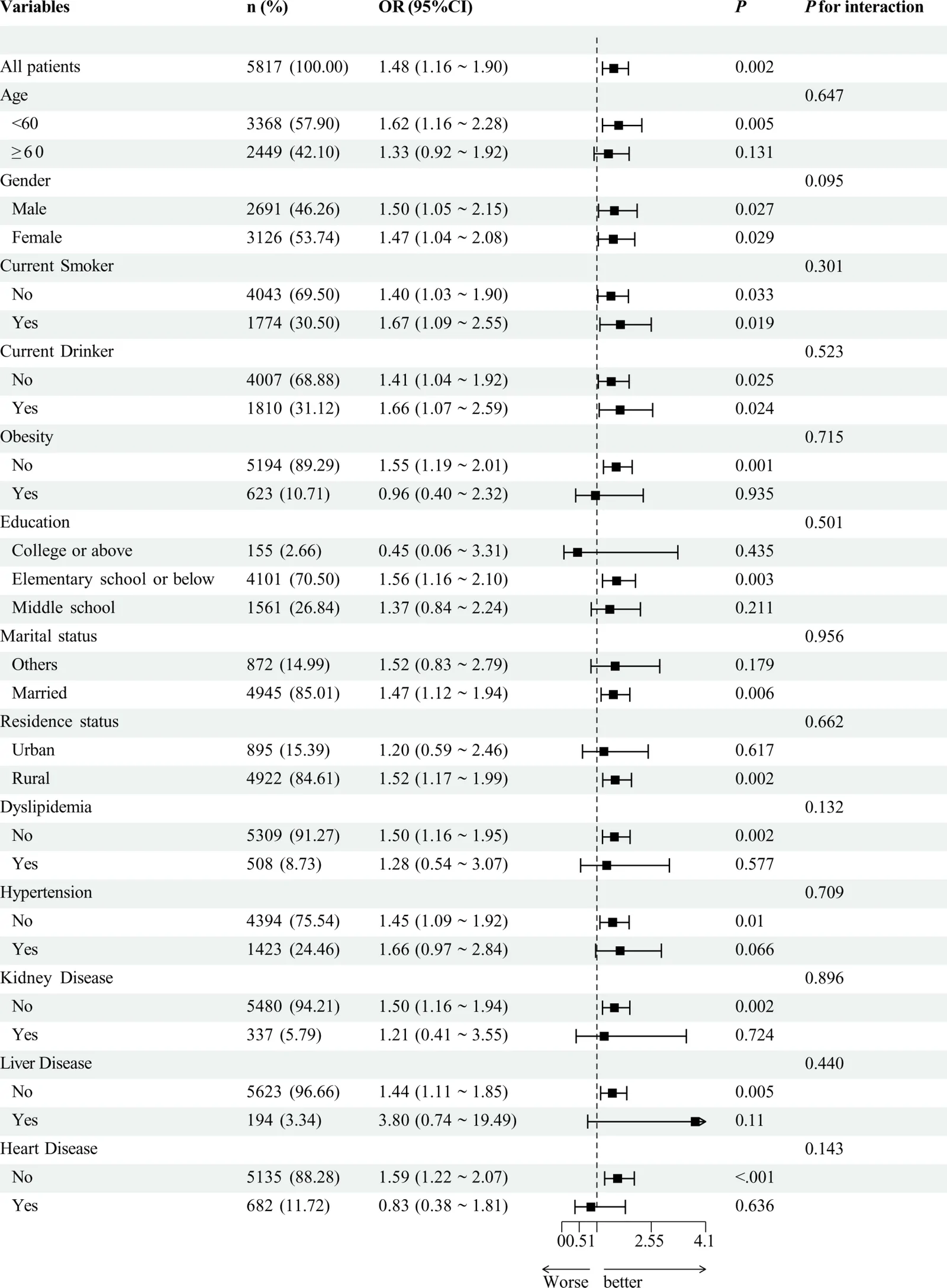
Subgroup analysis between cumRCII and DM incidence. CI = confidence interval, cumRCII = cumulative remnant cholesterol inflammation index, DM = diabetes mellitus.

### 3.5. Sensitivity analysis

First, the positive correlation between the cumRCII and incident DM was consistent after removing participants with any missing covariate values in the fully adjusted model (OR = 1.65, 95% CI = 1.26–2.16, Q2; OR = 1.92, 95% CI = 1.48–2.50, Q3; OR = 2.48, 95% CI = 1.92–3.20, Q4; OR = 1.24, 95% CI = 1.16–1.33, per 1 SD increase; Table 3). Second, after excluding individuals who had already developed DM at wave 2 (2013), the result aligned with the primary analysis (OR = 1.54, 95% CI = 1.18–2.01, Q2; OR = 1.86, 95% CI = 1.43–2.41, Q3; OR = 2.40, 95% CI = 1.87–3.09, Q4; OR = 1.25, 95% CI = 1.17–1.33, per 1 SD increase; Table 4). Third, we excluded prediabetic individuals in 2011, which yielded consistent results (OR = 1.60, 95% CI = 1.06–2.40, Q2; OR = 2.15, 95% CI = 1.46–3.18, Q3; OR = 2.20, 95% CI = 1.48–3.28, Q4; OR = 1.24, 95% CI = 1.14–1.40, per 1 SD increase; Table 5). Fourth, the results including participants with values beyond 3 standard deviations are consistent with the primary analysis (OR = 1.63, 95% CI = 1.25–2.12, Q2; OR = 1.88, 95% CI = 1.46–2.43, Q3; OR = 2.56, 95% CI = 2.00–3.29, Q4; OR = 1.20, 95% CI = 1.10–1.31 per 1 SD increase; Table 6). Finally, based on the results of model 4, the *E*-value calculated was 2.52 for DM.

**Table 3:** Association between the cumulative RCII and DM incidence after excluding individuals with any missing value.

Variables	Model 1		Model 2		Model 3		Model 4	
	OR (95% CI)	*P* value	OR (95% CI)	*P* value	OR (95% CI)	*P* value	OR (95% CI)	*P* value
CumRCII								
Per SD increase	1.28 (1.20–1.37)	**<.001**	1.27 (1.19–1.36)	**<.001**	1.27 (1.19–1.36)	**<.001**	1.24 (1.16–1.33)	**<.001**
Quartiles								
Q1	1.00 (reference)		1.00 (reference)		1.00 (reference)		1.00 (reference)	
Q2	1.71 (1.31–2.23)	**<.001**	1.69 (1.29–2.20)	**<.001**	1.69 (1.29–2.20)	**<.001**	1.65 (1.26–2.16)	**<.001**
Q3	2.09 (1.61–2.70)	**<.001**	2.05 (1.58–2.65)	**<.001**	2.05 (1.58–2.65)	**<.001**	1.92 (1.48–2.50)	**<.001**
Q4	2.80 (2.18–3.60)	**<.001**	2.72 (2.11–3.50)	**<.001**	2.71 (2.10–3.48)	**<.001**	2.48 (1.92–3.20)	**<.001**
*P*_for trend_		**<.001**		**<.001**		**<.001**		**<.001**

Model 1: crude.

Model 2: adjusted for age, gender.

Model 3: adjusted for age, gender, education level, marital status, smoking status, drinking status, residence, BMI.

Model 4: adjusted for age, gender, education level, marital status, smoking status, drinking status, residence, BMI, hypertension, dyslipidemia, liver disease, kidney disease, heart disease.

Bold values indicate statistically significant differences.

BMI = body mass index, CI = confidence interval, cumRCII = cumulative remnant cholesterol inflammation index, DM = diabetes mellitus, OR = odds ratio, RCII = remnant cholesterol inflammation index, SD = standard deviation.

**Table 4:** Association between the cumulative RCII and DM incidence after excluding individuals with DM at wave 2.

Variables	Model 1		Model 2		Model 3		Model 4	
	OR (95% CI)	*P* value	OR (95% CI)	*P* value	OR (95% CI)	*P* value	OR (95% CI)	*P* value
CumRCII								
Per SD increase	1.28 (1.20–1.37)	**<.001**	1.27 (1.19–1.36)	**<.001**	1.27 (1.19–1.36)	**<.001**	1.25 (1.17–1.33)	**<.001**
Quartiles								
Q1	1.00 (reference)		1.00 (reference)		1.00 (reference)		1.00 (reference)	
Q2	1.60 (1.22–2.08)	**<.001**	1.57 (1.21–2.05)	**<.001**	1.57 (1.20–2.04)	**<.001**	1.54 (1.18–2.01)	**.002**
Q3	1.98 (1.53–2.56)	**<.001**	1.95 (1.51–2.53)	**<.001**	1.95 (1.51–2.53)	**<.001**	1.86 (1.43–2.41)	**<.001**
Q4	2.67 (2.08–3.42)	**<.001**	2.61 (2.03–3.35)	**<.001**	2.60 (2.02–3.33)	**<.001**	2.40 (1.87–3.09)	**<.001**
*P*_for trend_		**<.001**		**<.001**		**<.001**		**<.001**

Model 1: crude.

Model 2: adjusted for age, gender.

Model 3: adjusted for age, gender, education level, marital status, smoking status, drinking status, residence, BMI.

Model 4: adjusted for age, gender, education level, marital status, smoking status, drinking status, residence, BMI, hypertension, dyslipidemia, liver disease, kidney disease, heart disease.

Bold values indicate statistically significant differences.

BMI = body mass index, CI = confidence interval, cumRCII = cumulative remnant cholesterol inflammation index, DM = diabetes mellitus, OR = odds ratio, RCII = remnant cholesterol inflammation index, SD = standard deviation.

**Table 5:** Association between the cumulative RCII and DM incidence after excluding prediabetic individuals at wave 1.

Variables	Model 1		Model 2		Model 3		Model 4	
	OR (95% CI)	*P* value	OR (95% CI)	*P* value	OR (95% CI)	*P* value	OR (95% CI)	*P* value
CumRCII								
Per SD increase	1.28 (1.15–1.44)	**<.001**	1.27 (1.13–1.42)	**<.001**	1.27 (1.13–1.42)	**<.001**	1.24 (1.11–1.40)	**<.001**
Quartiles								
Q1	1.00 (reference)		1.00 (reference)		1.00 (reference)		1.00 (reference)	
Q2	1.65 (1.10–2.48)	**.015**	1.63 (1.09–2.44)	**.018**	1.62 (1.08–2.43)	**.019**	1.60 (1.06–2.40)	**.024**
Q3	2.38 (1.62–3.50)	**<.001**	2.34 (1.60–3.44)	**<.001**	2.28 (1.55–3.36)	**<.0001**	2.15 (1.46–3.18)	**<.001**
Q4	2.47 (1.67–3.65)	**<.001**	2.40 (1.62–3.56)	**<.001**	2.39 (1.61–3.54)	**<.001**	2.20 (1.48–3.28)	**<.001**
*P*_for trend_		**<.001**		**<.001**		**<.001**		**<.001**

Model 1: crude.

Model 2: adjusted for age, gender.

Model 3: adjusted for age, gender, education level, marital status, smoking status, drinking status, residence, BMI.

Model 4: adjusted for age, gender, education level, marital status, smoking status, drinking status, residence, BMI, hypertension, dyslipidemia, liver disease, kidney disease, heart disease.

Bold values indicate statistically significant differences.

BMI = body mass index, CI = confidence interval, cumRCII = cumulative remnant cholesterol inflammation index, DM = diabetes mellitus, OR = odds ratio, RCII = remnant cholesterol inflammation index, SD = standard deviation.

**Table 6:** Association between the cumulative RCII and DM incidence without 3SD outlier exclusion.

Variables	Model 1		Model 2		Model 3		Model 4	
	OR (95% CI)	*P* value	OR (95% CI)	*P* value	OR (95% CI)	*P* value	OR (95% CI)	*P* value
CumRCII								
Per SD increase	1.29 (1.19–1.39)	**<.001**	1.26 (1.16–1.37)	**<.001**	1.23 (1.13–1.34)	**<.001**	1.20 (1.10–1.31)	**<.001**
Quartiles								
Q1	1.00 (reference)		1.00 (reference)		1.00 (reference)		1.00 (reference)	
Q2	1.58 (1.24–2.03)	**<.001**	1.66 (1.28–2.17)	**<.001**	1.66 (1.28–2.17)	**<.001**	1.63 (1.25–2.12)	**<.001**
Q3	2.00 (1.58–2.54)	**<.001**	2.01 (1.55–2.59)	**<.001**	2.01 (1.55–2.59)	**<.001**	1.88 (1.46–2.43)	**<.001**
Q4	2.95 (2.35–3.71)	**<.001**	2.80 (2.19–3.58)	**<.001**	2.80 (2.19–3.58)	**<.001**	2.56 (2.00–3.29)	**<.001**
*P*_for trend_		**<.001**		**<.001**		**<.001**		**<.001**

Model 1: crude.

Model 2: adjusted for age, gender.

Model 3: adjusted for age, gender, education level, marital status, smoking status, drinking status, residence, BMI.

Model 4: adjusted for age, gender, education level, marital status, smoking status, drinking status, residence, BMI, hypertension, dyslipidemia, liver disease, kidney disease, heart disease.

Bold values indicate statistically significant differences.

BMI = body mass index, CI = confidence interval, cumRCII = cumulative remnant cholesterol inflammation index, DM = diabetes mellitus, OR = odds ratio, RCII = remnant cholesterol inflammation index, SD = standard deviation.

## 4. Discussion

In this nationwide cohort, the cumRCII is significantly associated with incident DM among middle-aged and elderly Chinese adults. Notably, the relationship between the cumRCII and the incidence of DM exhibited a positive, nonlinear pattern. Subgroup analysis and sensitivity analysis further confirmed the robustness of the primary associations.

Lipid metabolism disorders and inflammation are risk factors for DM. Evidence from previous studies proved that elevated RC levels are more positively related to DM, prediabetes, and insulin resistance.^[32–34]^ Ma et al reported that elevated RC was an independent risk factor for hyperuricemia in 6447 participants, an association partially mediated by hypertension and inflammation.^[35]^ Crucially, Shao et al, leveraging both CHARLS and the English Longitudinal Study of Ageing, identified that long-term exposure to high RC levels and cumulative RC were significant risk factors for incident DM, independent of LDL-C levels.^[32]^ A study has demonstrated the role of elevated hsCRP in the development of DM.^[36]^ Several studies have revealed a notable association between hsCRP and DM or insulin resistance.^[8,37,38]^

Despite these findings, clinical evidence quantifying the combined impact of RC and systemic inflammation in populations remains limited. Therefore, our study calculated the RCII, a novel composite biomarker that involves RC and hsCRP to evaluate the synergistic interaction between lipids and inflammatory,^[27]^ and was proven to be associated with diverse diseases. Several recent studies have established the role of RCII in health problems. Chen et al found that elevated levels of RCII, both at baseline and cumulatively, were significantly associated with an increased risk of stroke.^[27]^ Further reinforcing its broad impact, Wang et al demonstrated that higher RCII levels were associated with increased all-cause mortality in middle-aged and elderly populations in the United States and China, with J-shaped or linear relationships observed for cause-specific mortality in the US cohort.^[25]^ Elevated RCII may indicate a mismatch between metabolic stress and immunomodulation, which is a proposed hypothesis requiring further mechanistic studies to confirm these biological pathways. Previous studies indicated a robust association between RCII and stroke.^[26,27]^ And it was revealed that higher RCII was linked with an elevated risk of cardiovascular disease.^[24]^ A prior investigation observed that the RCII significantly correlated with all-cause and cause-specific mortality.^[25]^ Restricted studies have examined the association between RCII and DM risk. Recent research has revealed a notable association between RCII and the risk of DM.^[39]^

Consistent with prior reports, utilizing data from CHRALS, we revealed that elevated RCII levels were significantly associated with an increased risk of DM. Several prior studies confirmed that elevated RC is associated with DM risk, yet they failed to integrate residual lipid burden and chronic inflammation. Furthermore, most previous studies relied on single RCII measurements, whereas our study introduces the novel concept of cumRCII exposure. Single-time point RCII only reflects the instantaneous lipid-inflammatory status at a single survey wave, ignoring its cumulative metabolic–inflammatory effects, and risking regression-dilution bias and impacting the accuracy of the results.^[40]^ The cumRCII accounts for long-term burden and variability, potentially reducing regression dilution bias and offering a more accurate risk assessment compared to single baseline measurements. In addition, a positive, nonlinear dose–response relationship was identified between the cumRCII and the incidence of DM. Fully adjusted logistic regression results demonstrated that cumRCII yielded a larger effect for incident DM compared with single RCII, proving cumulative exposure provided incremental value over single measurements.

The fully adjusted OR indicates that participants with the highest long-term lipid-inflammatory burden carry nearly 1.5 times higher odds of DM risk. This magnitude of association is comparable to well-recognized classic DM risk factors. In addition, RCS analysis identified 2 clear cutoff thresholds of RCII and cumRCII, which may serve as preliminary reference thresholds for clinical risk stratification. Furthermore, we observed that none of the subgroups exhibited a significant modification to the association between the cumRCII and DM incidence, which manifests the applicability of our findings to most individuals. The robust results of sensitivity analysis and *E*-value support the clinical reliability of effect size. The RCII may potentially reflect the interaction between residual lipoprotein accumulation and subclinical inflammation.

Traditional TC, LDL-C, HDL-C, and TG are the core lipid indicators for cardiovascular risk, but their association with DM is limited. Isolated hsCRP only reflects systemic inflammation without lipid metabolic information, while RC alone cannot reflect inflammatory status. The cumRCII further integrates RC and inflammation, capturing synergistic metabolic damage. In the full adjustment model, the cumRCII maintained a significant association with DM incidence, which means cumRCII adds extra information beyond conventional risk factors. Especially, the cumRCII reflects the cumulative effects of an individual on the physiological and pathological states represented by RCII over a corresponding period. Our findings fill the evidence gap regarding long-term RCII exposure associated with DM onset, suggesting its potential as an early biomarker for identifying individuals at elevated risk of DM.

Several limitations should be considered when interpreting these findings. First, the possibility of reverse causation cannot be entirely excluded. Participants with undiagnosed prediabetes or insulin resistance at baseline might have already exhibited elevated RCII levels due to early metabolic dysregulation. In this scenario, elevated RCII would be a consequence rather than a cause of the diabetic process. In addition, RCII may simply serve as a marker of shared underlying pathological processes, such as obesity or systemic insulin resistance, rather than playing a direct etiological role. Further longitudinal studies with repeated measures of insulin sensitivity are needed to disentangle these temporal relationships. To mitigate the bias from reverse causation by undiagnosed prediabetes, we performed a sensitivity analysis that excluded prediabetic individuals in 2011. The positive association between cumRCII and incident DM remained statistically robust, which partially weakens the interference of reverse causation from early glucose impairment. But this analytical adjustment cannot completely eliminate residual reverse causal bias.

There are several limitations in this study. First, the accuracy of DM diagnosis may be compromised due to the lack of oral glucose tolerance tests. Self-reported physician-diagnosed DM may lead to a potential memory bias. The absence of oral glucose tolerance testing likely caused underdiagnosis of DM, potential misclassification of prediabetes at baseline, and a lack of information on DM onset timing. Second, the potential for residual confounding exists. We used the *E*-value to quantify the potential implications of unmeasured confounders and found that an unmeasured confounder was unlikely to explain the entirety of the DM risk. The *E*-value indicated that our findings are relatively robust. Third, the calculation of cumRCII relied on only 2 biomarker measurements over 4 years, which assumes a linear and stable exposure trajectory. This may not fully capture the temporal variability of lipid and inflammatory markers. Fourth, there is potential for survivor bias and attrition bias, as participants who were lost to follow-up or died before wave 3 were excluded from the analysis, which may have skewed the results. Moreover, considering the potential introduction of selection bias by excluding many participants, our analysis cohort represents a relatively healthy and more stable population after comparing the baseline characteristics of included and excluded individuals. Consequently, the observed association between cumRCII and incident DM might be conservatively underestimated rather than inflated. Lastly, this is a study from Chinese middle-aged and elderly adults, which limits the generalizability of the findings to younger populations or other ethnic groups. The findings lack external validation in independent cohorts, which is necessary to confirm the robustness of the cumRCII metric.

## 5. Conclusion

The findings indicated that increased cumRCII exposure is significantly associated with higher DM risk in Chinese middle-aged and older adults. These findings suggest that cumRCII may serve as a potential risk marker for identifying individuals at higher metabolic risk, warranting further investigation.

## Acknowledgments

The authors thank the CHARLS participants and team.

## Author contributions

**Formal analysis:** Lianguang Xie, Xuan Zhou.

**Writing – original draft:** Lianguang Xie, Xuan Zhou.

**Writing – review & editing:** Chun Yao, Diankui Shui.


