# Supplementary material for: Cumulative exposure to the remnant cholesterol inflammation index and incident diabetes mellitus in middle-aged and elderly Chinese adults: A nationwide cohort study from CHARLS
Source: Medicine (Baltimore). 2026 Oct 9;105(41):e51063. doi: 10.1097/MD.0000000000051063 (PMC13657519; doi:10.1097/MD.0000000000051063)
Supplement: Supplementary file 1 [file medi-105-e51063-s001.docx]

**Table S1 Baseline characteristics of included and excluded participants**

| Variables | Total  (n = 17708) | Excluded participants  (n = 11891) | Included participants  (n = 5817) | *P* |  |
| --- | --- | --- | --- | --- | --- |
|  |  |  |  |  |  |
| Age, years | 58.50 ± 10.17 | 58.52 ± 10.82 | 58.44 ± 8.73 | 0.606 |  |
| BMI, kg/m^2^ | 24.16 ± 35.80 | 24.14 ± 35.53 | 24.18 ± 36.24 | 0.956 |  |
| SBP, mmHg | 129.58 ± 21.56 | 130.48 ± 21.84 | 128.08 ± 21.00 | **<0.001** |  |
| DBP, mmHg | 75.48 ± 12.26 | 75.80 ± 12.26 | 74.95 ± 12.23 | **<0.001** |  |
| HbA1c, % | 5.26 ± 0.82 | 5.42 ± 1.07 | 5.10 ± 0.39 | **<0.001** |  |
| FBG, mg/dL | 110.30 ± 37.33 | 120.58 ± 49.41 | 100.01 ± 11.58 | **<0.001** |  |
| TG, mg/dL | 134.91 ± 110.26 | 148.82 ± 135.95 | 120.95 ± 73.64 | **<0.001** |  |
| TC, mg/dL | 192.97 ± 38.89 | 193.89 ± 40.82 | 192.04 ± 36.84 | **0.010** |  |
| HDL-c mg/dL | 50.84 ± 15.33 | 49.59 ± 15.60 | 52.10 ± 14.94 | **<0.001** |  |
| LDL-c, mg/dL | 115.99 ± 34.91 | 115.11 ± 36.30 | 116.88 ± 33.44 | **0.006** |  |
| CRP, mg/L | 2.80 ± 7.63 | 3.49 ± 9.70 | 2.11 ± 4.62 | **<0.001** |  |
| Drinking status, n(%) |  |  |  | **0.011** |  |
| Never Drinker | 4862 (28.76) | 3131 (28.04) | 1731 (30.16) |  |  |
| Former Drinker | 1417 (8.38) | 959 (8.59) | 458 (7.98) |  |  |
| Current Drinker | 10628 (62.86) | 7078 (63.38) | 3550 (61.86) |  |  |
| Drinking status, n(%) |  |  |  | **0.206** |  |
| Never Drinker | 5380 (30.67) | 3573 (30.46) | 1807 (31.10) |  |  |
| Former Drinker | 1443 (8.23) | 994 (8.47) | 449 (7.73) |  |  |
| Current Drinker | 10718 (61.10) | 7163 (61.07) | 3555 (61.18) |  |  |
| Education level, n(%) |  |  |  | **<0.001** |  |
| College or above | 818 (4.62) | 663 (5.58) | 155 (2.66) |  |  |
| Elementary school or below | 11771 (66.53) | 7670 (64.59) | 4101 (70.50) |  |  |
| Middle school | 5103 (28.84) | 3542 (29.83) | 1561 (26.84) |  |  |
| Gender, n(%) |  |  |  | **0.003** |  |
| Male | 8478 (47.88) | 5787 (48.68) | 2691 (46.26) |  |  |
| Female | 9228 (52.12) | 6102 (51.32) | 3126 (53.74) |  |  |
| Marital status, n(%) |  |  |  | **<0.001** |  |
| Others | 3526 (19.93) | 2654 (22.34) | 872 (14.99) |  |  |
| Married | 14170 (80.07) | 9225 (77.66) | 4945 (85.01) |  |  |
| Residence status, n(%) |  |  |  | **<0.001** |  |
| Urban | 3969 (22.47) | 3074 (25.95) | 895 (15.39) |  |  |
| Rural | 13694 (77.53) | 8772 (74.05) | 4922 (84.61) |  |  |
| Dyslipidemia, n(%) |  |  |  | **<0.001** |  |
| No | 15517 (90.15) | 10286 (89.57) | 5231 (91.31) |  |  |
| Yes | 1696 (9.85) | 1198 (10.43) | 498 (8.69) |  |  |
| Hypertension, n(%) |  |  |  | **0.001** |  |
| No | 12935 (74.04) | 8558 (73.29) | 4377 (75.56) |  |  |
| Yes | 4535 (25.96) | 3119 (26.71) | 1416 (24.44) |  |  |
| Kidney disease, n(%) |  |  |  | 0.383 |  |
| No | 16473 (94.39) | 11025 (94.50) | 5448 (94.17) |  |  |
| Yes | 979 (5.61) | 642 (5.50) | 337 (5.83) |  |  |
| Liver disease, n(%) |  |  |  | 0.389 |  |
| No | 16833 (96.53) | 11236 (96.45) | 5597 (96.70) |  |  |
| Yes | 605 (3.47) | 414 (3.55) | 191 (3.30) |  |  |
| Heart disease, n(%) |  |  |  | 0.179 |  |
| No | 15337 (87.81) | 10222 (87.57) | 5115 (88.28) |  |  |
| Yes | 2130 (12.19) | 1451 (12.43) | 679 (11.72) |  |  |
